# The Infectious Diseases Orchestrator: Embracing AI Literacy in the Agentic Era

**DOI:** 10.1093/ofid/ofaf794

**Published:** 2025-12-26

**Authors:** John J Hanna, Richard J Medford

**Affiliations:** Information Services, ECU Health, Greenville, North Carolina, USA; Department of Internal Medicine, ECU Brody School of Medicine, Greenville, North Carolina, USA; Clinical Informatics Center, University of Texas Southwestern, Dallas, Texas, USA; Department of Internal Medicine, University of Texas Southwestern, Dallas, Texas, USA; Information Services, ECU Health, Greenville, North Carolina, USA; Department of Internal Medicine, ECU Brody School of Medicine, Greenville, North Carolina, USA; Clinical Informatics Center, University of Texas Southwestern, Dallas, Texas, USA; Department of Internal Medicine, University of Texas Southwestern, Dallas, Texas, USA

**Keywords:** AI agents, artificial intelligence, infectious diseases practice, large language models, natural language processing

## Abstract

Artificial intelligence (AI) is rapidly transforming healthcare, with agentic AI systems positioned to perceive, reason, and act within clinical environments. For infectious diseases (ID) clinicians, agentic AI presents both opportunity and imperative; to embrace AI literacy and remain actively engaged in shaping their design rather than becoming passive adopters in clinical care, antimicrobial stewardship, and infection control. Historical examples show that professions failing to adapt to automation faced challenges, highlighting the urgency for ID specialists to understand AI's evolving role. While AI can streamline documentation, surveillance, and decision support, clinicians must advocate for high-quality data, define appropriate automation boundaries, and ensure human oversight in critical decisions. ID communities should lead efforts to educate clinicians, establish AI governance policies in ID operational practices, and foster interdisciplinary collaboration to guide responsible AI integration. AI literacy is the “no-regret” investment that will enable clinicians to lead this transformation—ensuring that AI supports, augments, and, when appropriate, automates the repetitive, searchable, and time-consuming tasks. The future of ID practice will be defined by how effectively clinicians leverage AI to enhance care, promote equitable access, and reclaim time for the human dimensions of medicine.

## Teaser

Infectious diseases faces a critical juncture as agentic artificial intelligence (AI) systems emerge, bringing both promise and the risk of passive adoption. This article calls for proactive AI literacy and provides strategies for ID specialists to prepare for responsible integration, while urging ID communities to lead through education, interdisciplinary collaboration, and governance.

Infectious diseases (ID) practice is entering an inflection point as artificial intelligence (AI) advances from predictive tools to agentic systems that can perceive, reason, and act within clinical environments [1–4]. These capabilities are increasingly incorporated within electronic health record (EHR) ecosystems [5] to streamline decision-making processes, enhance documentation efficiency, and provide real-time clinical insights.

These rapidly evolving AI technologies signal a fundamental shift in how medicine—and particularly ID—will be practiced. Unlike many other specialties that rely on well-defined clinical pathways, ID practice is inherently data-intensive, decision-heavy, and context-dependent, often requiring real-time synthesis of epidemiology, microbiology, antimicrobial resistance patterns, and detailed clinical risk factors to make optimal patient care decisions. The very nature of ID work—navigating diagnostic uncertainty, responding to outbreaks, managing antimicrobial stewardship programs, and adapting to emerging pathogens—aligns closely with AI's strengths in processing large datasets, identifying patterns, and augmenting clinical reasoning.

However, leveraging AI effectively in ID requires more than just passive adoption; it demands that ID clinicians become orchestrators of AI agents, actively guiding their use in patient care. Many AI-powered tools are already emerging in clinical workflows today, such as antimicrobial resistance deterministic algorithms [6–8], sepsis predictive models [9, 10], ambient listening for automated clinical documentation [11–14], and computer vision-assisted hand hygiene monitoring [15, 16]. Yet, many examples of AI implemented in healthcare to date introduce challenges, including frequent high false positive rates that contribute to alert fatigue [17], poor performance across populations or in external validation [18], omission and commission errors in AI-generated text that create documentation inaccuracies [19], and algorithmic biases that can perpetuate inequities if used uncritically [20, 21]. Without foundational AI literacy, ID clinicians risk becoming mere consumers of these tools rather than active orchestrators who can interpret, validate, and optimize their use.

This perspective argues that AI literacy is now an essential core competency for ID specialists. While we recognize marked heterogeneity in data infrastructure, governance, and workforce capacity across settings, from tertiary health systems to rural hospitals and resource-constrained clinics. Against a backdrop of persistent ID workforce shortages and burnout, agentic workflows can convert repetitive, searchable, and time-consuming tasks into supervised, machine-assisted processes that return time to clinicians. Equally important, these workflows offer a path to democratize access to ID expertise through hub-and-spoke service models, mobile-first designs, and lightweight edge agents that extend high-quality decision support to smaller US facilities and to low- and middle-income countries.

AI literacy—understanding what these systems can and cannot do, how they perform, and how to supervise them—is the no-regret investment that enables ID clinicians to set boundaries on automation, ensure equity and safety, and lead the next phase of technology-enabled care.

## From deterministic rule-based systems to AI agents, an evolution story

AI in healthcare has evolved through major phases, each expanding its capacity to support clinical reasoning transitioning from deterministic rule-based systems to advanced agentic AI systems that dynamically coordinate multiple models and automation tools with or without a human in the loop [22].

Rule-based systems operationalized expert logic through deterministic if-then pathways [23, 24], enabling structured alerts to flag drug interactions [25, 26], trigger allergy checks [27, 28], and enforce antimicrobial prescribing guidelines [29–31]. While effective for enforcing standard best practices, they lack adaptability and struggle with context-dependent decisions [32, 33].

Machine learning introduced pattern recognition and trained classifiers across large, structured datasets [34, 35], powering predictive models for outcomes like sepsis [17]. Yet, these models often faltered in generalizability, external validation, and integration within workflows while accounting for unstructured data [17, 18].

To bridge that gap and process unstructured data, such as clinical notes [36–38], radiology reports [39–41], and unstructured comments in microbiology lab results [42, 43], natural language processing (NLP) techniques were introduced. Despite progress, rule-based and statistical NLP systems faced limitations in scalability, accuracy, and computational cost [44, 45].

The recent introduction of Large Language Models (LLMs) marked a breakthrough, enabling systems to generate human-like responses, summarize complex information, and engage in conversational interactions [46–48]. Unlike earlier NLP, advanced LLMs can reason across complex contexts [49, 50], and already power ambient documentation [51], automated chart summarization [52], and AI-assisted patient communication [53–55].

 

### What are AI Agentic Systems and What Makes Them Transformational?

The latest leap in AI development is the rise of agentic AI systems [1–4], which combine LLM capabilities with automation, real-time data retrieval, and task execution. These systems move beyond text generation—they are programmed to plan, act, and adapt dynamically [56, 57]. When applied in healthcare [2, 58, 59], unlike classic LLMs integration that require explicit prompts [52, 53], AI agents can act dynamically, responding to real-time inputs and coordinating across multiple systems.

**Figure 1.**
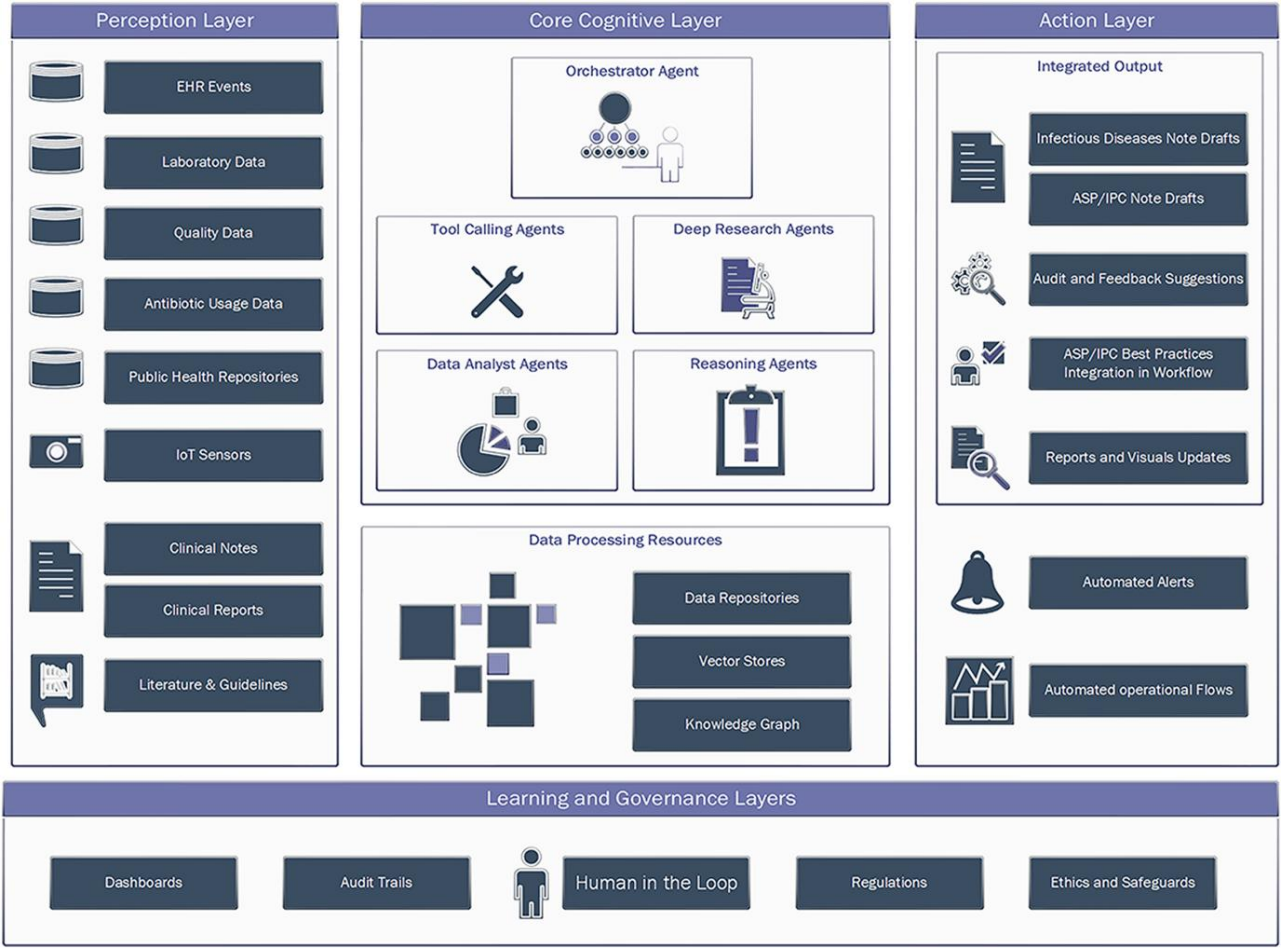
Example AI agentic system architecture in healthcare settings to support infectious diseases, infection prevention and control (IPC), and antimicrobial stewardship (ASP) operations.

An agentic AI architecture typically comprises several key components [56, 57] (Figure 1):

- Perception: The layer that gathers data from the environment through sensors, databases, or real-time inputs. In healthcare settings, these inputs could include everything from patient vital signs, laboratory results, and clinical notes to external data such as public health repositories or camera feeds. The goal is to acquire a comprehensive understanding of the current state to inform subsequent actions.
- Cognition: Once data are collected, the system processes and interprets them using AI. including LLMs and predictive analytics models that analyze patterns, identify correlations, and infer potential outcomes. In healthcare, this could mean predicting patient risk, identifying potential diagnoses, or analyzing treatment effectiveness based on data.
- Planning: With the insights gained from the cognition layer, agentic AI systems develop strategies to achieve defined objectives. This involves considering potential outcomes, weighing the pros and cons of different actions, and selecting the most optimal path forward. For instance, the system might plan an antimicrobial stewardship intervention by scheduling timely audits, recommending alternative treatments, and optimizing operational resource allocation.
- Action: After planning, the system moves on to execute the planned tasks. This involves interacting with other systems, automation tools, or real-world environments to carry out actions. In clinical practice, this could mean automatically adjusting a patient's medication, coordinating with healthcare staff for infection control measures, drafting clinical documentation, or alerting key stakeholders.
- Learning: Agentic AI systems can continuously improve performance by learning from past interactions and adapting to new information. This learning process involves revisiting previous actions, assessing their effectiveness, and incorporating new data and experiences to refine future decision-making processes.

Examples range from autonomous self-driving vehicles, which integrate real-time data from multiple sensors to navigate roads and avoid obstacles without human intervention, to computer using agents [60] that interact with graphical user interfaces using virtual browser; like website buttons, menus, and text fields that are visible on a screen.

### How Agentic AI Systems Could Transform Health Systems and Infectious Diseases Practice

The emergence of agentic AI systems has the potential to fundamentally change how healthcare organizations operate. ID clinicians, who work at the intersection of diagnostics, antimicrobial stewardship, infection prevention, and public health, may experience particularly profound shifts in their daily workflows if these technologies become widely integrated.

As health systems begin integrating agentic AI systems into clinical workflows, ID clinicians need to prepare for a fundamental shift in how they interact with technology. The traditional approach—where clinicians spend significant time gathering, interpreting, and documenting data—could gradually give way to an AI-augmented model, where clinicians spend more time interacting with the patients, while guiding, supervising, and refining AI-driven processes rather than manually executing every step themselves.

Different operational ID roles could experience change in distinct ways (examples in Table 1). ID consultative care demands rapid synthesis of evolving microbiology, imaging, procedures, and prior treatment histories. Example supported workflows include assembling source-linked summaries, generating draft assessments, and candidate differentials for clinician review while accounting for patients’ context, organizational resources, and guidelines. Such agentic workflows can extend high-quality ID expertise access to resource-restrained settings lacking on-site ID expertise.

**Table 1.** Example Agentic Workflows that Could Support Different Tasks by ID Subdomain

ID Subdomain	Example Tasks/Workflows That Could Be Supported By Agentic AI
Consultative care (including immunocompromised hosts)	Pre-round data assembly with source links; dynamic differential drafts; continuous watch for new positives/deterioration
Antimicrobial stewardship	Continuous bug–drug mismatch detection; IV→PO opportunities; de-escalation; duration tracking with policy-aware suggestions
Infection prevention and control	Isolation adherence checks; early cluster detection from EHR/sensor signals; auto-compiled evidence packets; dynamic reporting
Outpatient parenteral antimicrobial therapy	Lab/level monitoring; adherence support; device complication surveillance; risk-based escalation to ID team
Virtual ID care	E-consult triage; multilingual communication; outside-record summarization; automated follow-up scheduling, between-visits asynchronous communication support and coaching
Research and quality improvement	Autonomous research loops, auto-phenotyping; registry curation; QI dashboards with drift checks; study feasibility screens
Education & training	Point-of-care micro-tutorials; simulated consult cases with feedback; documentation coaching, presentation preparation
Continuity of care (HIV, chronic wound care, etc.)	Longitudinal trend tracking; gap-in-care flags; coordination with community resources
Public health	Pre-adjudication of reportable events/criteria; data harmonization; automated situation reports with audit trails

Stewardship teams today rely on manual audits, rule-based alerts, and retrospective chart reviews to assess antimicrobial use. With agentic AI, continuous monitoring of antimicrobial prescribing patterns, real-time resistance trends, and patient-specific risk factors might allow for AI-driven recommendations and automated real-time modifications. When this occurs, stewardship teams will be able to shift from individual case interventions to supervising AI-driven antimicrobial optimization across entire patient populations.

**Figure 2.**
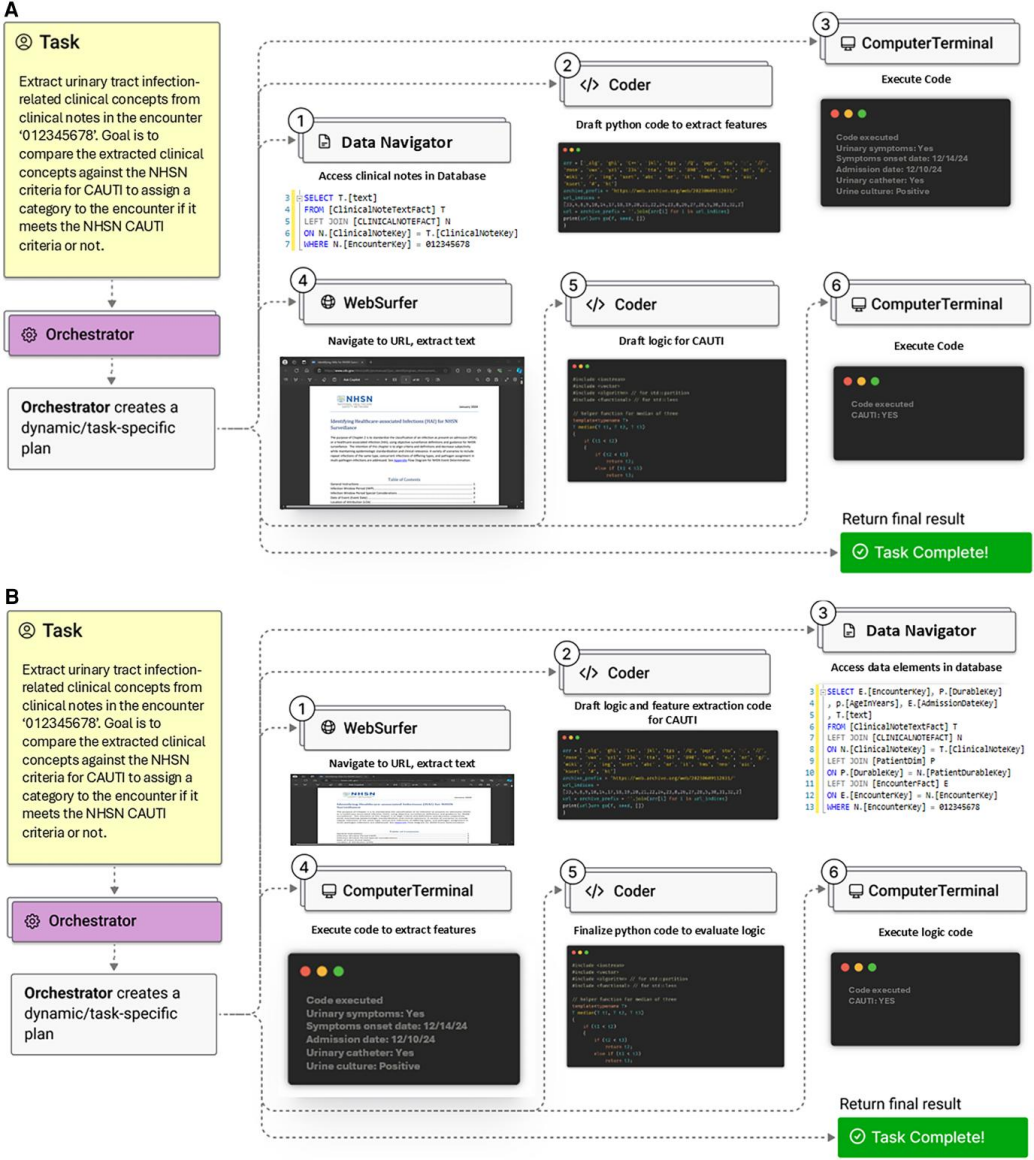
Example Agentic AI system in action. Reimagined from Magentic-One: A Generalist Multi-Agent System for Solving Complex Tasks [61]. (*A*) Agentic AI evaluating NHSN CAUTI criteria against an encounter. (*B*) Agentic AI returning a report for all admission encounters with active vancomycin and piperacillin-tazobactam on the medication list including other relevant risk factors.

When it comes to infection prevention, instead of manually tracking compliance with hand hygiene, isolation protocols, and environmental cleaning, AI agents could autonomously analyze camera feeds, sensor data, and EHR interactions to support enforcement of infection control protocols. As these systems advance, infection preventionists may transition from data collection and compliance tracking to higher-level risk assessment and intervention planning based on AI-driven insights (Figure 2).

Similarly, supporting outpatient parenteral antimicrobial therapy (OPAT), ambulatory, and HIV care workflows like monitoring required labs, drug levels, and adherence can all be subject to augmentation through agentic workflows. Virtual ID care can also be supported by agentic triage systems, multi-language assistance, and expanding episodic care delivery into one patient journey.

Even research and quality improvement tasks are frequently repetitive, searchable, and time consuming. From literature review to reasoning over hypothesis and execution on planned analysis while keeping logs for humans in the loop, semi-autonomous research loops can accelerate discovery and the continuous improvement cycles while lowering the entry barrier to resource-constrained settings.

These anticipated shifts require a proactive approach to AI literacy, workflow adaptations, and new decision-making frameworks in a technology-driven environment. Responsible agentic workflow implementations could turn the current reactive and siloed care delivery environment into one of continuous, data-driven coordination where the ID physician becomes the orchestrator.

### The Critical Need for AI Literacy Among Infectious Diseases Clinicians

History is filled with examples of roles that resisted technological evolution and became obsolete. Telephone operators, assembly-line workers, and travel agents each saw their roles transformed or replaced by automation. A similar shift is occurring in healthcare. While highly skilled clinical roles are unlikely to disappear, the way they interact with technology is rapidly changing. Routine administrative, documentation and analytical tasks are increasingly being handled by AI and automation, and ID workflows are no exception. The challenge for ID clinicians is not whether AI will play a role in our field—but how we will integrate it into our expertise.

Agentic AI systems are actively demonstrating their ability to automate structured, rule-based processes. As these technologies advance, many roles that depend on predictable workflows and repetitive decision-making will likely face major transformation. For example, AI-driven automation is already replacing human roles such as medical scribes and documentation specialists where ambient listening is being used to generate notes and automate downstream workflows. Similarly, AI agents have the potential to automate many of the manual data analysis tasks of reporting analysts and abstractors.

Rather than resistance to change, ID specialists have the opportunity to shape how AI integrates into their practice by identifying where human expertise is indispensable. ID clinicians can shape the future of AI in healthcare by advocating for higher quality data pipelines specific to infectious diseases care, defining when automation is appropriate and when human intervention is necessary for specific tasks, and identifying opportunities for AI to simplify complex, repetitive, and time-consuming tasks that they perform frequently.

### Balancing Benefits, Risks, and Mitigation

Gains in timeliness and coverage must be matched by safeguards for validity and trust. While AI models are only as good as the data they learn from or have access to, many health systems suffer from inconsistent documentation, fragmented patient records, and poorly defined clinical concepts, which can lead to unreliable AI outputs. ID clinicians should collaborate with data scientists and clinical informaticists to standardize ID-related definitions, antimicrobial use metrics, and acceptable diagnostic criteria when automation is deemed appropriate. ID clinical expertise is going to be paramount in evaluating the accuracy of unstructured-to-structured data transformations and AI-generated text on patients with infectious diseases.

Automated decision-making can amplify disparities unless explicitly monitored. ID expertise can support high-quality bias-free datasets that accurately reflect different patient populations for training and minimize disparities in AI-driven decision-making. An AI literate workforce can also design equity checks across all the AI lifecycle stages for solutions integrated in ID practices. The ID orchestrators can be valuable assets to AI governance councils to define transparency, privacy, and autonomy scope boundaries.

In the future of agentic AI automation, not all aspects of ID care should be automated. The experience of clinical microbiology laboratories offers a guiding analogy. Despite heavy regulations, labs achieved near-total automation not through wholesale replacement but through staged literacy, rigorous data standards, and continuous oversight. ID should follow a similar trajectory.

ID clinicians should take the lead in determining which tasks can be fully automated, which tasks require a human-in-the-loop approach, and where AI should serve as an augmentation tool. Simple, well-defined tasks—such as flagging inappropriate antibiotic prescriptions or detecting missing infection control documentations—may be safely handled by AI as first use cases. Complex clinical decisions, such as managing infections in immunocompromised patients with atypical presentations, are likely to continue to benefit from human judgement, even if AI provides task support. AI augmented workflow may use AI to assist in summarizing data, highlighting risks, and making preliminary assessments, but final decision-making would remain clinician driven.

### Preparing ID Clinicians for the AI Era, the Role of Professional Societies

ID communities—the collective ecosystem of ID professionals, societies, academic programs, and leadership bodies that shape practice standards, education, and policy within the field—must take an active role in ensuring that ID clinicians are equipped with the necessary AI literacy. While individual clinicians need to adapt and engage with AI-driven workflows, professional societies are uniquely positioned to provide guidance, education, policy advocacy, and workforce development to help ID specialists navigate this transformation. To support the transition, ID communities should focus on three key areas (Table 2) over the next few years: education and training, fostering interdisciplinary collaboration, and responsible AI governance and policy development within ID.

- AI literacy needs to become a core competency for ID clinicians, just as epidemiology, microbiology, and antimicrobial stewardship have long been foundational to the field. ID communities should establish structured educational programs to help clinicians develop a working knowledge of AI, its applications, and its limitations. This can be through AI-focused workshops and courses, online educational modules, mentorship programs, and published clinical guidelines on AI use in ID. These should focus on introducing ID specialists to the fundamentals of AI, AI performance evaluation, AI-generated recommendation interpretation, and AI integration in ID workflows. The overall goal is for ID specialists to gain trust and confidence. Not trust and confidence in AI, but trust in understanding how these AI tools work, and confidence in knowing how to use them acknowledging their potential and limitations.
- AI development and implementation require expertise beyond traditional ID training, including knowledge of data science, informatics, and health system operations. ID communities should take the lead in facilitating interdisciplinary collaboration between ID clinicians, AI researchers, policymakers, and health system leaders. To achieve this, The ID communities should establish AI interest groups and committees to guide AI research and implementation in ID practice, promote AI-focused research funding to encourage ID specialists to engage with AI-driven systems, create partnerships with AI developers and health technology companies to ensure AI tools are clinically validated, ethically designed, and aligned with real-world ID challenges, and facilitate cross-disciplinary conferences and publications encouraging ID specialists to collaborate with AI and informatics experts in shaping the future of AI in ID.
- As AI adoption increases, ID communities should work to establish clear principles for AI use in ID practice areas, emphasizing transparency, accountability, and patient safety. Key governance initiatives may include position statements on appropriate AI use in ID care and operations, regulatory guidance on the use of AI in infection prevention and antimicrobial stewardship programs, and best practices for AI oversight in ID workflows.

**Table 2.** Key Priorities for Advancing AI Literacy and Responsible AI Integration in ID

Focus Area	Frontline Providers	ID Divisions/health Systems	ID Training Programs	ID Professional Societies
Education and training	Develop baseline AI literacy as a core competency, similar to microbiology or stewardship. Participate in AI-focused CME, workshops, or online modules.	Support continuing education programs and protected time for AI learning. Integrate AI literacy into departmental training and clinical decision support workflows.	Incorporate AI fundamentals, ethics, and evaluation into ID curricula Develop mentorship opportunities in AI-related research and QI projects.	Publish clinical guidelines and position papers on AI literacy in ID. Curate accessible educational resources (courses, webinars, workshops, toolkits.)
Interdisciplinary collaboration	Engage with data scientists, informaticians, and health IT teams when using AI-driven tools. Provide clinical insights to improve AI design and relevance	Establish cross-disciplinary federated AI learning communities or interest groups. Partner with AI developers and industry to co-design and validate tools aligned with clinical needs.	Encourage trainee participation in interdisciplinary projects linking ID, AI, and informatics. Promote joint training tracks or electives in data science, digital health, and clinical informatics.	Facilitate national working groups connecting ID and AI experts. Advocate for AI-focused research funding and joint conferences.
Governance and policy development	Apply ethical principles when using AI in patient care (transparency, accountability, and patient safety) Report and discuss AI performance issues through institutional channels	Develop policies for safe AI deployment in infection prevention and antimicrobial stewardship operations. Create overarching AI governance structure	Teach frameworks for AI ethics, regulation, and responsible implementation. Include governance case studies in training.	Issue position statements on AI use in ID care Develop best practice guidelines and advocate for clear regulatory standards.

Augmentation and redesign—not replacement—are the pragmatic pathways forward [62]. The next few years will be pivotal in determining how AI reshapes ID practice. While today's ID challenges center on shortages, burnout, and widening access gaps, the near-term opportunity lies in converting repetitive or readily searchable tasks into supervised automation that expands capacity and reduces variance. Agentic AI adds a new layer of opportunity: to multiply human expertise, democratize access globally, and restore time for the human dimensions of care

### Conclusion

The era of agentic AI in healthcare is approaching, and ID clinicians stand at a critical crossroads. While the extent to which AI can augment and automate repetitive, searchable, and time-consuming tasks remains uncertain, those who fail to integrate AI into their expertise risk being unprepared. By embracing AI literacy, defining AI's role in clinical decision-making and operational workflows, and advocating for responsible AI integration, ID specialists can lead rather than follow in this transformation. The question is no longer whether AI will impact ID practice—but how well ID clinicians will adapt and shape its future. To support this transition, ID communities should promote AI education and training, interdisciplinary collaboration, and responsible AI governance and policy development within ID.
